# Clg2p interacts with Clf and ClUrase to regulate appressorium formation, pathogenicity and conidial morphology in *Curvularia lunata*

**DOI:** 10.1038/srep24047

**Published:** 2016-04-04

**Authors:** Tong Liu, Yuying Wang, Bingchen Ma, Jumei Hou, Yazhong Jin, Youli Zhang, Xiwang Ke, Lianmei Tai, Yuhu Zuo, Kishore Dey

**Affiliations:** 1 Institute of Plant Pathology and Applied Microbiology, School of Agronomy, Heilongjiang Bayi Agricultural University, Daqing, Heilongjiang, 163319, P. R. China; 2 State Key Laboratory of Crop Stress Biology in Arid Regions, Northwest A & F University, Yangling, Shanxi, 712100, P. R. China; 3 National Coarse Cereals Engineering Research Center, Daqing, Heilongjiang, 163319, P. R. China; 4 Department of Plant and Environmental Protection Sciences, University of Hawaii at Manoa, 3190 Maile Way, Honolulu, HI, 96822, USA

## Abstract

Ras is a small GTPase that regulates numerous processes in the cellular development and morphogenesis of many organisms. In this study, we identified and functionally characterized the *Clg2p* gene of *Curvularia lunata*, which is homologous with the Ras protein. The *Clg2p* deletion mutant (Δ*Clg2p*) had altered appressorium formation and conidial morphology and produced fewer, smaller lesions compared with the wild-type strain. When a dominant *Clg2p* allele was introduced into the mutant, all of these defective phenotypes were completely restored. To further understand the regulation of *Clg2p* in appressorium formation and conidial morphology, and its role in pathogenicity, seven Clg2p-interacting proteins were screened using a yeast two-hybrid assay. Two of these proteins, Clf, a homologue of Mst11, which corresponds to MAP kinase kinase kinase in *Magnaporthe oryzae*, and urate oxidase (designated ClUrase) were functionally characterized. Clg2p specifically interacted with Clf through its RA domain to regulate appressorium formation and pathogenicity, whereas the Clg2p-ClUrase interaction regulated conidial morphology without affecting fungal pathogenicity. This report is the first to elucidate the regulatory mechanism of the key Ras protein Clg2p in *C. lunata.*

*Curvularia* leaf spot caused by *Curvularia lunata* (Wakker) Boed was responsible for one of most devastating diseases of corn in China during the 1990 s^1^,^2^. It is currently one of most widely distributed corn leaf diseases worldwide, resulting in substantial yield losses^3^,^4^,^5^,^6^. The occurrence of various pathogenic types of *C. lunata* and its potential to evolve into other pathogenic strains has generated a global interest among scientists to uncover the molecular mechanism of its virulence and pathogenicity^7^,^8^,^9^,^10^,^11^,^12^. Understanding the molecular mechanism could reveal effective control strategies for this disease.

*C. lunata* mainly infects the leaves, sheaths and husks of corn plants. The initial yellow, water-soaked spots progress to round, spindle or oval shapes in later stages of infection. The distinctive features of this pathogen are dark brown conidiophores with characteristic boomerang or knee-like shapes on top. The conidia are crescent-shaped^13^. During the infection process, conidia of this pathogen land on the host plant and adhere to the leaf surface. Upon receiving appropriate stimuli during the initial host-surface recognition, signals are relayed for germ tube extension and production of infection structures, which then germinate to produce infection hyphae^14^,^15^. The fungus also secretes lytic enzymes, which aid in penetrating the plant cell wall^16^. The germ tube may also differentiate into an appressorium after receiving appropriate physical or chemical signals from plant leaf surfaces, such as hydrophophobicity, topography or surface hardness, and then it uses mechanical force to penetrate the host plant. In *C. lunata*, the melanin present in the appressoria is also believed to reinforce mechanical strength during infection by maintaining turgor pressure^17^. The mechanism of penetration of *C. lunata* is similar to *Magnaporthe oryzae*^18^, but appressoria are not essential for penetration.

The interplay of signals between the host and pathogenic fungi is believed to be mediated by Ca^2+^, cyclic adenosine monophosphate (cAMP) and mitogen-activated protein kinase (MAPK)^19^,^20^,^21^. In the past decade, MAPK has been increasingly implicated in regulating a host of functions in the fungus ranging from the development of hyphal growth, appressorium formation, maturation, invasive growth, conidiation and pathogenicity^22^,^23^. For example, the *Mst11-Mst7-Pmk1* MAPK cascade regulates appressorium formation and infectious growth of *Magnaporthe grisea*^24^. In *Cochliobololus heterostrophus, Chk1* is involved in appressorium formation, female fertility and full virulence^25^. However, to date, only two homologues of MAPK, *Clk* and *Clm1,* have been identified in *C. lunata. Clk* is important for vegetative growth, biosynthesis of cell wall-degrading enzymes and pathogenicity^10^. The *Clm1* mutant impairs fungus cell wall formation and conidial morphology, reduces conidiospore production, and lowers disease symptoms on corn leaves^26^. Although these findings expand our knowledge of the conidiation, cell wall formation and pathogenicity of *C. lunata*, the underlying molecular mechanisms regulating these processes are still not well understood. In our previous study, we created a normalized, full-length cDNA library of *C. lunata* and annotated a number of expressed sequence tag (EST) sequences using bioinformatics^27^. Among these ESTs, we identified a Ras homologue (designated *Clg2p*) from *C. lunata* and investigated its biological and regulatory role during infection.

The Ras protein family belongs to a class of small GTPases that are important organizers of signal transduction mechanisms due to their direct involvement in intracellular signal transduction pathways^28^,^29^,^30^,^31^,^32^,^33^,^34^. Ras proteins also affect cellular signal transduction pathways with important regulatory roles in morphogenesis, conidiation, appressorium development and pathogenicity in fungi^35^,^36^,^37^,^38^. For example, in *Saccharomyces cerevisiae*, Ras proteins elevate intracellular cAMP levels and trigger a kinase that results in sensitivity to heat shock, nutrient starvation, and inhibition of sporulation^35^. In *Fusarium graminearum*, the Ras2 protein reportedly affected the Gpmk1 MAP kinase pathway, regulating growth and pathogenicity of the fungus^36^. The Ras2 protein in *Magnaporthe grisea* is involved in appressorium formation and pathogenicity^37^. The Ras family homologous protein StRas2 in *Setosphaeria turcica* plays an important role in morphogenesis, conidiation, and appressorium development^38^. Little is known, however, about the role of Ras proteins in *C. lunata*.

The results presented in this study show that the Clg2p protein interacts with Clf and ClUrase, the urate oxidase protein, to regulate appressorium development and conidial morphology. This interaction is required for full pathogenicity in *C. lunata*. These results provide new insights into the signal transduction mechanism during the infection process of *C. lunata* and may lead to optimal target sites for chemical control of this devastating pathogen.

## Results

### Cloning and characterization of *Clg2p*

**Figure 1:**
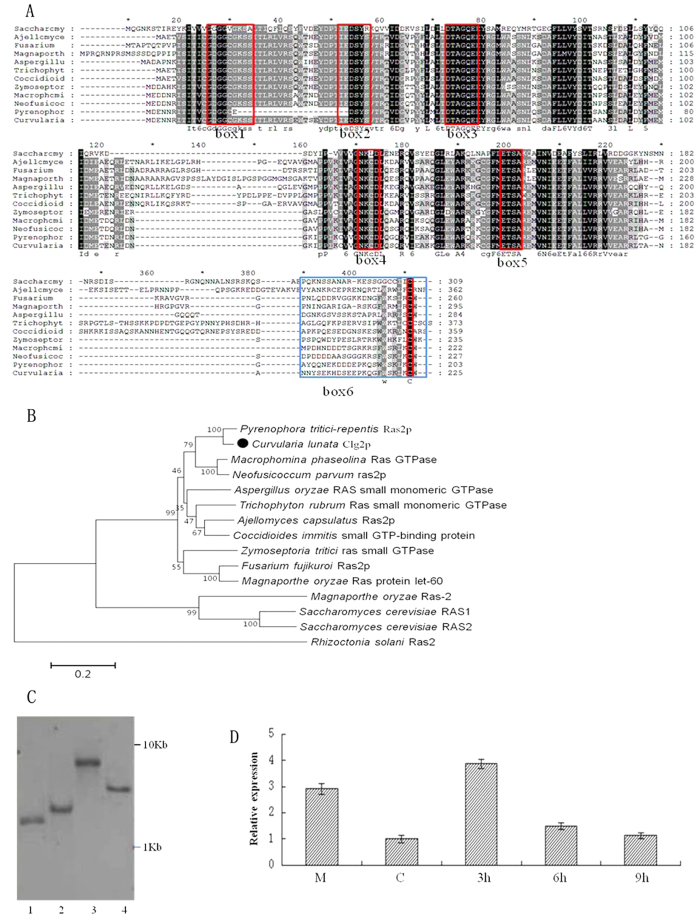
Molecular characteristics of *Clg2p* in *C. lunata.* (**A**) Analysis of conserved domains of *Clg2p*. Box1, box3, box4 and box5 are the GTP/GDP domains; box2 is a binding site for downstream effector molecules (RA); and box6 is a variable region containing a CAAX motif. (**B**) A phylogenetic tree representing the phylogenetic relationships of Clg2p proteins and Ras proteins among related fungi. The number for each interior branch was the percentage of the bootstrap value (1000 replicates). (**C**) Analysis of copy number of *Clg2p* in the genome. A 951-bp PCR fragment amplified with primers 1F/R as a template of strain CX-3 DNA was labelled using Biotin to make the probe. (**D**) Expression patterns of *Clg2p* by qRT-PCR. M and C (x-axis) represent mycelial growth in potato dextrose (PD) medium for 3 d and conidia collected from 7 d culture on PDA plates at 28 °C in the dark, respectively. On the graph, 3 h, 6 h and 9 h (x-axis) represent germinating conidia collected from cellophane overlaid on PDA plates at 25 °C after 3, 6 and 9 h of growth, respectively. The error bars were calculated based on three replicates.

Among the various clones sequenced from the cDNA library, a particular sequence of 480 bp showed high similarity with *Ras2p* gene sequences of *Pyrenophora tritici-repentis* Pt-1C-BFP, indicating that it was most likely a partial sequence of *Ras2p*. This sequence has been designated *Clg2p*. Subsequent RACE reactions yielded a 797 bp fragment and a 175 bp fragment at the 3′-end and 5′-end, respectively. The complete full-length *Clg2p* contained a 678 bp open reading frame (ORF), a 61 bp 5′-untranslated region (5′-UTR) and a 549 bp 3′-UTR with a poly(A) tail. The ORF of *Clg2p* encoded a 225 amino acid protein with a molecular weight (MW) of 25.5 kDa and a calculated isoelectric point of 4.98. Using the ScanProsite tool, the encoded Clg2p protein was found to include 4 highly conserved GTP/GDP domains, a binding domain for the downstream effector molecule (RA), and a CAAX motif in the COOH-terminus, similar to other Ras proteins (Fig. 1A). A phylogenetic tree of *Clg2p* constructed using the neighbour-joining method indicated a close relationship with *Ras2p* from *P. tritici-repentis* Pt-1C-BFP (Fig. 1B). Analysis of the gene structure showed that *Clg2p* has four exons (53, 51, 386 and 176 bp) and three introns (58, 149 and 78 bp) within the 951 bp sequence (Fig. S1). Southern analysis identified a single copy of Clg2p in the *C. lunata* genome (Fig. 1C). The quantitative PCR (Q-PCR) analysis indicated that the expression level of Clg2p was significantly different at different developmental stages. The expression was highest in 3 h germinating conidia, indicating that the expression of *Clg2p* correlates with the pathogen infection, growth and appressorium development (Fig. 1D).

### Targeted deletion and complementation of *Clg2p*

**Figure 2:**
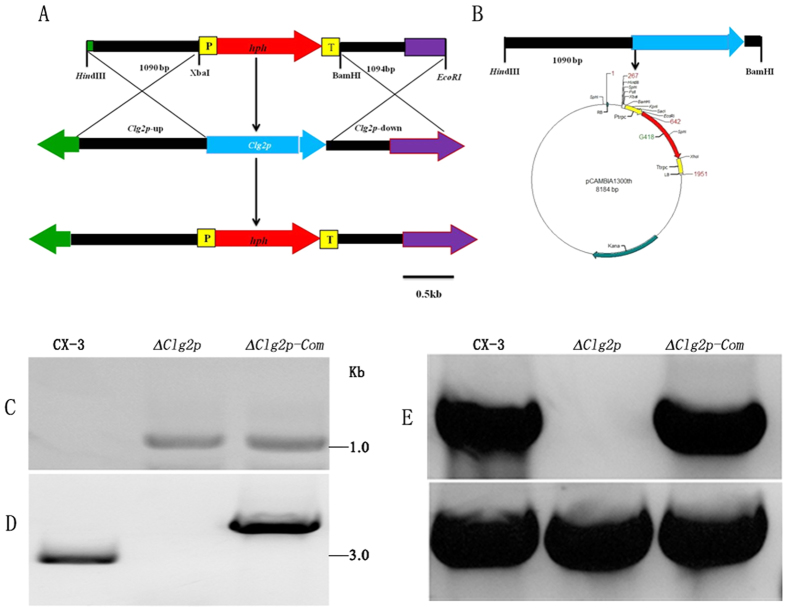
Construction and confirmation of the *Clg2p* deletion mutant and complementation strain. (**A**) Construction of the deletion vector lacking *Clg2p*. (**B**) Construction of the complementation vector of *Clg2p* as described in the methods section. (**C,D**) Southern blotting analysis of the *Clg2p* deletion mutant (Δ*Clg2p*) and complementation strain (*Clg2p-*Com). (**C**) Southern blotting was performed on isolated total genomic DNA from CX-3, Δ*Clg2p* and *Clg2p-*Com after digestion with *Xba*I and *Bam*HI. A 607-bp PCR fragment of *hph* was used as the hybridization probe. (**D**) A second Southern blotting analysis was performed using isolated total genomic DNA from wild-type strain CX-3, Δ*Clg2p* and *Clg2p-*Com after digestion with a single-cutter restriction enzyme, *Hin*dIII, using a 951-bp PCR fragment of *Clg2p* as the probe. (**E**) Clg2p expression in CX-3, Δ*Clg2p* and *Clg2p-*Com was analysed by RT-PCR using the *Clg2p* gene-specific primers clg2pds and clg2pda. A predicted 415-bp fragment was obtained from the wild-type CX-3 strain and the complemented strain but was absent in the Δ*Clg2p* mutant.

To generate the *Clg2p* deletion mutant, the *Clg2p* gene was replaced by the selective marker gene *hph* through ATMT transformation. The knockout construct was created by insertion 1090 bp upstream and 1094 bp downstream of *Clg2p* into the Δ1300 vector. This vector contains a selective marker gene, *hph*, flanked by the upstream and downstream sequences of the *Clg2p* ORF (Fig. 2A). The transformants were screened and selected on medium with 250 μg/ml hygromycin and verified by PCR for the presence of the resistance gene (*hph*). A genetic complementation strain (*Clg2p-*Com) was constructed by reintroducing the native *Clg2p* gene, including the 1.090-kb promoter region and DNA of *Clg2p* (Fig. 2B). The putative deletion mutants and genetic complementation strain were confirmed by Southern blotting (Fig. 2C,D) and RT-PCR (Fig. 2E). Finally, one deletion mutant and complementation strain were obtained and used for functional analysis in this study.

### The Clg2p gene affects appressorium formation and conidial morphology and is essential for its full pathogenicity

**Figure 3:**
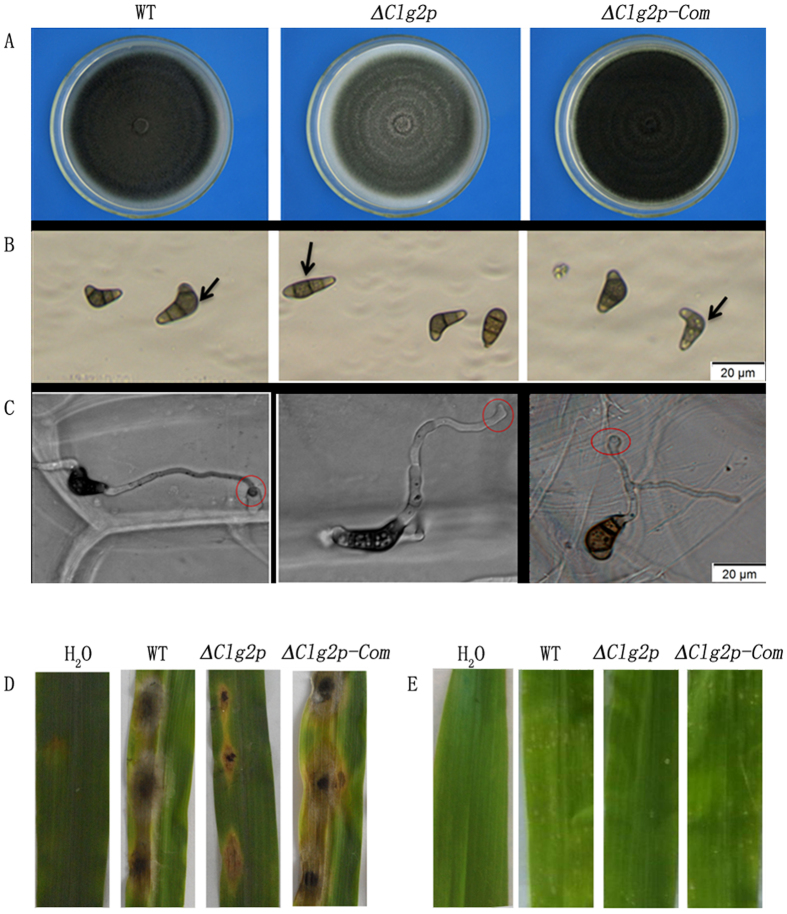
Functional analysis of the *Clg2p* gene. (**A**) The colony morphology of the wild-type strain CX-3 (WT), Δ*Clg2p* and *Clg2p*-Com cultured on PDA media for 7 d; (**B**) Abnormal conidial morphology was observed by microscopy, bar = 20 μm; (**C**) Abnormal appressoria were observed by microscopy, bar = 20 μm; (**D**,**E**) Disease symptoms on unwounded (**D**) leaves of maize inoculated with mycelial plugs from CX-3, Δ*Clg2p* and *Clg2p*-Com. Inoculation using agar plugs without fungus was used as a control. (**E**) Disease symptoms on susceptible corn leaves sprayed with conidial suspensions of CX-3, Δ*Clg2p* and *Clg2p*-Com. Inoculation with sterile distilled water containing 2% glucose and 0.02% Tween 20 was used as a negative control. Typical leaves were photographed 3 d after inoculation.

The results on colony morphology, mycelial vegetative growth, conidiation, conidia germination, conidial morphology and appressorium formation in the wild-type strain CX-3, the deletion mutant (Δ*Clg2p*), and the complementation strain (*Clg2p-*Com) as well as their effect on pathogenicity are presented in Fig. 3A and Table 1.

The most striking finding was the deformed and abnormal appressorium structure observed in Δ*Clg2p* (Fig. 3B). By contrast, the wild-type strain CX-3 and *Clg2p-*Com showed normal appressorium structure and position. When we examined conidiation and conidial morphology, we found that most of the conidia produced by Δ*Clg2p* were abnormal in appearance and mostly appeared as straightened conidia (Fig. 3C). However, the amount of conidia produced did not significantly change between Δ*Clg2p* and the ectopic transformant (Ect). *Clg2p-*Com also showed normal conidial morphology. The pathogenicity of the deletion mutant was analysed by infection assays in the susceptible corn inbred line Huangzao 4. The results indicated that the lesions on unwounded leaves caused by Δ*Clg2p* were smaller in shape than those caused by the wild-type strain CX-3 and *Clg2p-*Com (Fig. 3D). Moreover, in spray infection assays with 15 d old seedlings, numerous curvularia spots were observed on corn leaves sprayed with CX-3 or *Clg2p-*Com at 3 dpi, whereas fewer spots were observed on leaves sprayed with the Δ*Clg2p* under the same experimental conditions (Fig. 3E). These results clearly suggest that *Clg2p* is required for full pathogenicity in *C. lunata*. In addition, our results also showed that the *Clg2p* gene has a role in appressorium formation and position and in conidial morphology.

### Screening and identification of proteins associated with Clg2p

To understand the mechanism of Clg2p-mediated appressorium formation, conidial morphology and pathogenicity in *C. lunata*, we screened the constructed cDNA library of *C. lunata* for Clg2p-interacting proteins in a yeast two-hybrid (Y_2_H) assay. Before performing the Y_2_H assay, it was confirmed that pGBKT7-Clg2p was not activated automatically (Fig. S2) and does not have any toxic effects. The Y_2_H screen was performed twice on the cDNA library, and 7 proteins were obtained in total (Table S1). One of the Clg2p-interacting proteins is a homologue of MAPKKK, similar to Raf in *S. Cerevisiae* and Mst11 of the *Mst11-Mst7-Pmk1* MAPK cascades in *M. oryzae*; this protein was designated Clf in this study. Another interacting protein was identified is urase oxidase and named ClUrase. Other proteins identified included membrane-binding proteins and hypothetical proteins, which are listed in Table S1. The *Clf* and *ClUrase* genes were selected for further characterization. The ORFs of the *Clf* and *ClUrase* sequences were extracted from *C. lunata* genomic DNA and confirmed by RT-PCR. The structural characteristics of the Clf and ClUrase proteins are shown in Figs S3 and S4A,B.

### Interaction of Clg2p and Clf

**Figure 4:**
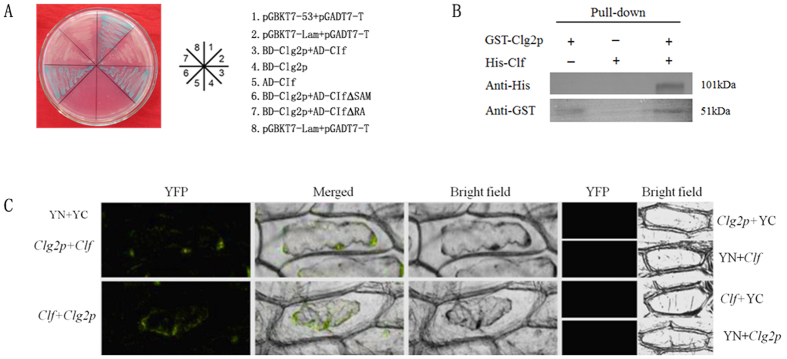
The interaction between Clg2p and Clf. **(A)** The interaction between Clg2p and Clf structural domains (RA and SAM) of Clf in yeast. Targeted H_2_Y with Clg2p as the bait and with the ORF, RA domain, or SAM domain of Clf as the prey. The interaction of pGBKT7-53 and pGADT7-T was used as the positive control, and the interaction of pGBKT7-Lam and pGADT7-T was used as the negative control. **(B)** A pull-down assay analysing the interaction between Clg2p and Clf. GST-Clg2p bound to GST beads were mixed with His-Clf purified from IPTG-induced *E. coli* BL21 (DE3). The bound proteins were eluted after removing the unbound proteins by washing and analysed by immunoblotting with anti-GST or anti-His antibodies. (**C)** Interaction between Clg2p and Clf in a plant cell. BiFC of Clg2p and Clf in transiently transformed onion epidermal cells. pspYNE-Clg2p+pspYCE, pspYNE+pspYCE-Clf, pspYNE-Clf+pspYCE and pspYNE+pspYCE-Clg2p were used negative controls. The YFP fluorescence signal was detected and observed using confocal microscopy.

To validate the interaction between Clg2p and Clf, we performed a targeted yeast two-hybrid (Y_2_H) assay, protein pull-down *in vitro* and a bimolecular fluorescence complementation (BiFC) assay. The results from the targeted yeast two-hybrid assay clearly showed that *Clg2p* physically interacted with *Clf*. In addition, the presence of a well-conserved Ras-association (RA) domain further supports the occurrence of such an interaction. Previous studies also indicated that such domains could interact with Ras-homologous proteins^37^, suggesting that the Clg2p protein may also interact with the RA domain of the Clf protein. Therefore, to further investigate whether the Clg2p protein interacts with the RA domain of Clf, we used a targeted yeast two-hybrid assay. The results shown in Fig. 4A clearly suggest that the Clg2p protein interacts with the RA domain of Clf but not the SAM domain of Clf.

For pull-down assays, the full length of Clg2p was expressed as a glutathione S-transferase (GST) fusion protein (GST-Clg2p) and was bound to a glutathione-agarose column. Additionally, Clf was also fused with the 6× His tag (His-Clf) and added to a glutathione-agarose column inoculated with GST-Clg2p, which was then eluted repeatedly with buffer to remove the non-specifically bound proteins. Proteins retained on the beads were eluted with buffer and resolved on SDS-PAGE (Fig. S5). The GST-Clg2p and His-Clf retained on the beads were detected by immunoblotting with anti-GST-tag and anti-His-tag antibodies, respectively. The results clearly suggest a direct interaction between the Clg2p and Clf proteins as shown in Fig. 4B. Furthermore, the BiFC assay allowed for visualization of the interactions to be mainly localized in the plasma membrane of the plant cells. As expected no YFP signals were observed in whole plant cells used as negative controls (Fig. 4C).

### Clg2p physically interacts with Clf via its RA domain to regulate appressorium formation, and Clg2p affects pathogenicity

**Figure 5:**
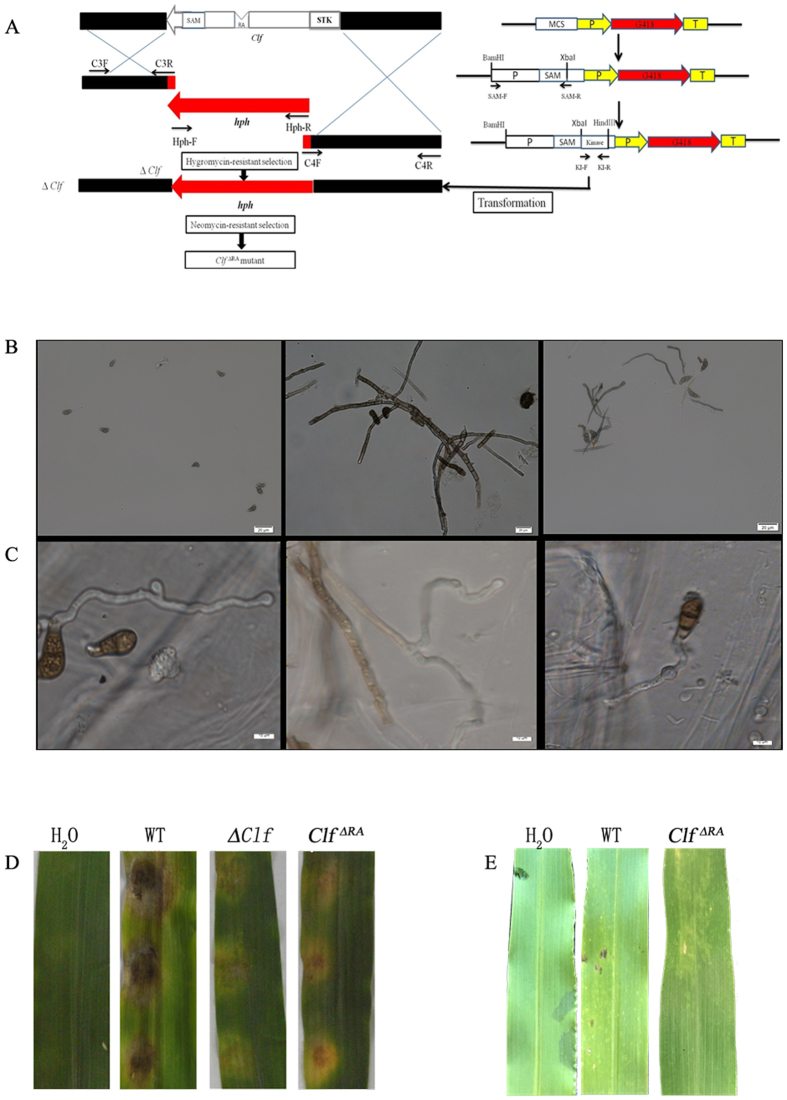
Conidiation, appressorium formation and pathogenicity of the *Clf* deletion and RA domain deletion mutants. (**A**) Construction of the *Clf* deletion and RA domain deletion mutants. (**B**) Abnormal conidial morphology was observed by microscopy, bar = 20 μm. (**C**) Abnormal appressorium structure and position were observed by microscopy, bar = 20 μm. (**D**) Leaves of maize inoculated by mycelial plugs from the wild-type strain CX-3, Δ*Clf* and *Clf*^ΔRA^. Inoculation using agar plugs without fungus was used as a control. (**E**) Disease symptoms on leaves of maize inoculated by conidial suspensions from the wild-type strain CX-3 and *Clf*^ΔRA^. Inoculation with sterile water containing 2% glucose and 0.02% Tween 20 was used as a negative control.

The above results indicated that Clg2p interacted with Clf, but only with the RA domain of Clf. To further investigate the functional relationship of Clg2p with Clf, we generated mutants with *Clf* ORF (Δ*Clf*) and *Clf*-RA domain deletions (*Clf*^ΔRA^) (Fig. 5A). The (Δ*Clf*) and *Clf*-RA domain deletion (*Clf*^ΔRA^) mutants were confirmed by Southern blotting (Fig. S6) and RT-PCR (Fig. S7). Interestingly, the Δ*Clf* deletion mutant of *C. lunata* did not produce conidia on PDA medium (Fig. 5B). Most exhibited abnormal morphology such as the lack of conidia production or loss of function such as lost capacity to infect maize leaves, similar to the phenotype of the *Clk* deletion mutant observed in *C. lunata*^19^. A swollen structure formed in the hyphal tips of Δ*Clf* (Fig. 5C), whereas, in the *Clf*^ΔRA^ mutant, most appressoria showed abnormal morphology and position compared with the wild-type strain CX-3 (Fig. 5C). Furthermore, smaller and fewer lesion areas were observed when either mycelial plugs or conidial suspensions of *Clf*^ΔRA^ mutants were sprayed to inoculate maize leaves than those observed when inoculated by the wild-type strain CX-3 (Fig. 5D,E). Overall, our results suggest that, in *C. lunata,* the interaction of *Clg2p* with *Clf* occurs via its RA domain, and this interaction is involved in appressorium formation, morphological development and pathogenicity.

### Clg2p interacts with ClUrase to regulate conidial morphology

**Figure 6:**
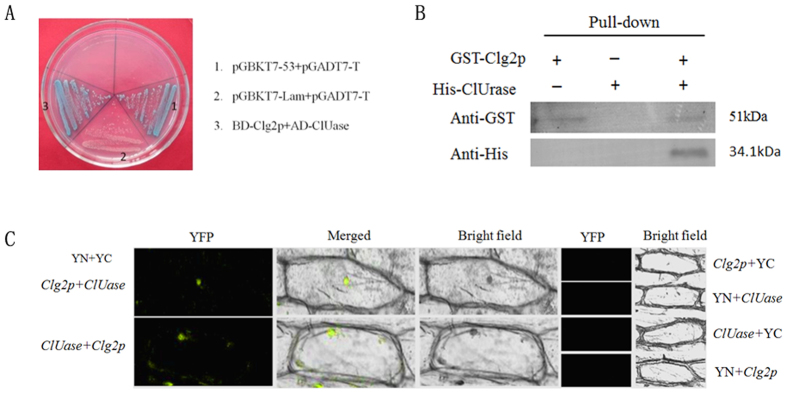
Clg2p interacts with ClUrase. **(A)** Clg2p interacts with ClUrase in yeast cells. The full-length gene *ClUrase* was used as bait and the full-length gene *Clg2p* was used as prey in targeted Y_2_H assays. (**B**) Clg2p directly interacts with ClUrase *in vitro*. In the pull-down assays, GST-Clg2p bound to GST beads was mixed with His-ClUrase purified from IPTG-induced *E. coli* BL21 (DE3). The bound proteins were eluted after removing unbound proteins by washing and analysed by immunoblotting with anti-GST or anti-His antibodies. (**C)** BiFC analysis of the Clg2p interaction with ClUrase. Clg2p and ClUrase were constructed with the N-terminal (YN) and C-terminal (YC) halves of YFP separately under the control of a CaMV35S promoter and were co-expressed transiently in onion epidermal cells. YFP expression was monitored by confocal laser scanning microscopy. pspYNE-Clg2p+pspYCE, pspYNE+pspYCE-ClUrase, pspYNE-ClUrase+pspYCE and pspYNE+pspYCE-Clg2p were used as negative controls. The YFP signal was observed using confocal microscopy.

To confirm whether Clg2p also directly interacts with ClUrase and, if so, to determine the location of their interactions, we performed the same set of assays as those described above. The results showed that a positive interaction between Clg2p and ClUrase in targeted Y_2_H assays (Fig. 6A). In the pull down assays, the His-ClUrase fusion protein was successfully induced to express in *E. Coli* BE3, and inoculated into the beads containing GST-Clg2p. After elution of non-specificially protein, the bait and prey protein were also eluted (Fig. S8), and subjected to Western blotting analysis. The results showed that the GST-Clg2p and His-Clf were detected by immuno-bloting with anti GST-tag and His-tag antibody respectively (Fig. 6B), confirming the interaction in-vitro between Clg2p and ClUrase. The BiFC assay further indicated that the interaction was mainly restricted to the cell membrane and the nucleus of the plant cell (Fig. 6C).

**Figure 7:**
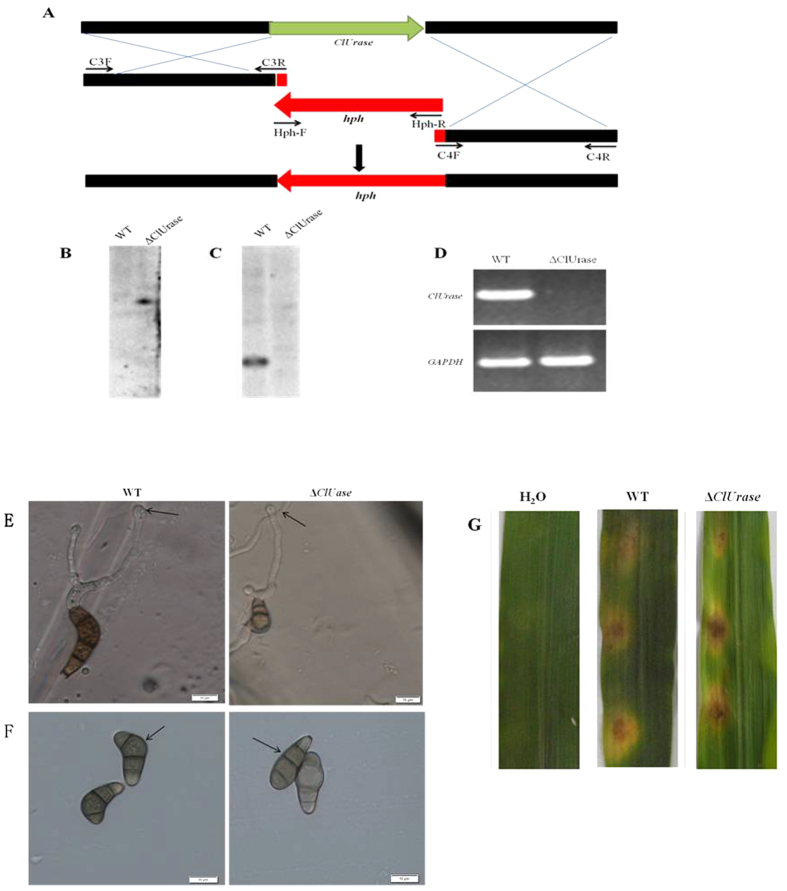
Construction and confirmation of the *ClUrase* deletion mutant and functional analysis of *ClUrase*. (**A**) Construction of the deletion vector for *ClUrase.* Southern blotting analysis of the *ClUrase* deletion mutant (Δ*ClUrase*). Total genomic DNA from CX-3 and Δ*ClUrase* were digested with the restriction enzyme *PaeI* and probed with a 610-bp fragment of *hph* (**B**). Southern blotting with a 688-bp PCR fragment of *ClUrase* used as the probe (**C**). RT-PCR analysis of *ClUrase* expression using the *ClUrase* gene-specific primers ClU-F and ClU-R in the wild-type strain CX-3 and Δ*ClUrase*. A predicted 654-bp fragment was found in the wild-type strain CX-3 but was absent in the Δ*ClUrase* mutant (**D**). Microscopic observation of appressorium formation and position, bar = 20 μm (**E**). Conidial morphology was observed by microscopy, bar = 20 μm (**F**). Leaves of maize inoculated with mycelial plugs from CX-3 and Δ*ClUrase*. Inoculation using agar plugs without fungus was used as a control (**G**).

To determine the functional relationship of the Clg2p and ClUrase interaction, we generated a *ClUrase* deletion mutant (designed Δ*ClUrase*) by transforming it in hygromycin-resistant protoplasts of *C. lunata* (Fig. 7A). PCR analysis from three of the transformants suggested that only one may be a *ClUrase* gene deletion mutant because it had the *hph* gene but lacked the *ClUrase* gene. Southern analysis (Fig. 7B,C) and RT-PCR (Fig. 7D) further confirmed that this transformant lacked the *ClUrase* gene compared with the wild type. Although Δ*ClUrase* had no obvious changes in colony morphology, vegetative growth, conidial yield or appressorium formation (Fig. 7E), we observed a clear change in the conidial morphology of the Δ*ClUrase* mutant, with approximately 47.23% uncurved conidia, compared with the wild-type strain (Fig. 7F and Table 1). To determine whether these abnormal conidia affected the pathogenicity of the organism, conidial suspensions of the wild-type CX-3 and Δ*ClUrase* strains were inoculated on unwounded maize leaves. Inoculation with ClUrase and the wild-type strain CX-3 resulted in the same size lesions, suggesting that the *ClUrase* disruption had little or no effect on pathogenicity (Fig. 7G). Therefore, our results suggest that the interaction of Clg2p with ClUrase apparently regulated the conidial morphology without affecting the pathogenicity of the pathogen.

## Discussion

**Figure 8:**
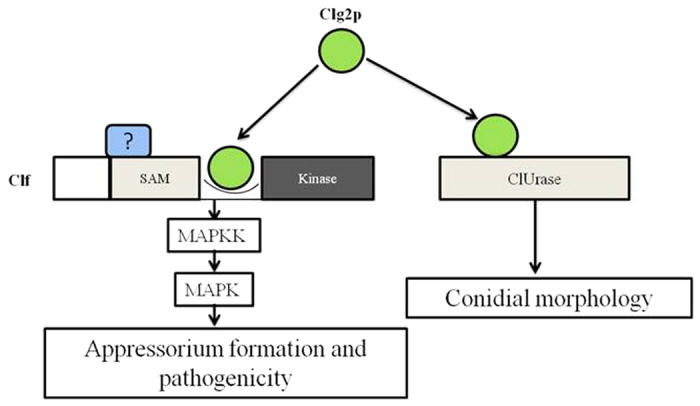
Hypothetical model of functions of Clg2p in *Curvularia lunata.* Clg2p is a Ras GTPase protein that interacts with the RA of MAPKKK (Clf). Clg2p acts with *Clf* to regulate the MAPK pathway and affects appressorium formation and pathogenicity in *Curvularia lunata*. Additionally, Clg2p interacts with ClUrase, which affects conidial morphology.

*C. lunata* is a phytopathogenic fungus that causes *Curvularia* leaf spot in maize. After a serious outbreak in the 1990 s, scientist achieved moderate success in controlling the fungus with the introduction of resistant maize cultivars. However, the disease has returned in recent years due to the continuous cultivation of these cultivars. This selection pressure is believed to have led to the evolution of the pathogen, which produces a highly virulent strain of *C. lunata*. Therefore, the pathogen poses a major threat to the agro-security of China^12^,^21^,^39^. A priority in the current corn breeding programme is to understand the variability in virulence and pathogenicity factors in the plant-pathogen interaction, which are closely related, and then to develop resistant cultivars^40^,^41^. To further this understanding, we developed a full-length cDNA library from the mycelium of *C. lunata*^27^. Bioinformatic analyses of the derived EST sequences identified a Ras homologous gene, *Clg2p*, with a close relationship to *Ras2p* from *P. tritici-repentis* Pt-1C-BFP. Initially, the expression pattern of *Clg2p* was highest during conidial germination, correlating its expression with pathogen infection and underscoring its potential role in fungal pathogenicity. To clarify the biological role of this gene during pathogenesis, we isolated the complete ORF and created its deletion mutant. The deletion mutant Δ*Clg2p* was compared with the wild-type strain and its complemented strain for conidial morphology, mycelial growth, conidiation, appressorium formation and pathogenicity on susceptible corn plants. The most striking effects of the *Clg2p* deletion were deformed, abnormal appressoria and conidia. When tested for pathogenicity, the mutant produced fewer, smaller lesions than those of the wild-type strain or its complemented strain. These effects were reversed when the native dominant gene was replaced in the mutant. This result suggested that *Clg2p* is an important component of a signal transduction pathway involved in the pathogenicity of *C. lunata*. Therefore, to identify the complete network of other interacting proteins of Clg2p, we used yeast two-hybrid screening. Two proteins in particular, *Clf* and *ClUrase*, were selected for further characterization based on the bioinformatics analysis. *Clf* was identified as a homologue of Mst11 from *M. oryzae*. A targeted yeast two-hybrid assay, protein pull-down *in vitro* and a bimolecular fluorescence complementation assay not only revealed that Clg2p interacted physically with *Clf* via its RA domain but also showed that the interaction was localized to the plasma membrane of the plant cell. Interestingly, the mutant generated for the domain (*Clf*^ΔRA^) did produce conidia but the appressoria developed from them were abnormal in appearance. In the pathogenicity assays, the mutants produced fewer, smaller lesions on inoculated leaves. These results were similar to those of Δ*Clg2p* described above. A functional analysis of ClUrase also revealed a physical interaction with Clg2p, which was restricted to the cell membrane and the nucleus. Moreover, the *ClUrase* mutant (Δ*ClUrase*) produced abnormally shaped conidia similar to Δ*Clg2p*. Unlike Δ*Clg2p*, however, the pathogenicity of Δ*ClUrase* was not affected. Overall, our results suggested that Clg2p interacted with *Clf* to regulate appressorium formation, morphology, and pathogenicity. However, Clg2p interacted with *ClUrase* to regulate conidial morphology without affecting the pathogenicity of *C. lunata* (Fig. 8).

The Ras protein is a key regulatory molecule and is well conserved across kingdoms. It acts as a switch by cycling between inactive and active GTP-bound forms. It plays an important role in controlling cell growth, proliferation, and morphogenesis^42^,^43^. Recently, there has been a growing interest in studying the role of Ras proteins in fungal development, morphogenesis, and pathogenesis^44^,^45^,^46^,^47^,^48^,^49^,^50^. In some well-studied plant fungal pathogens, such as *M. oryzae*^37^, *S. turcica*^38^ and *Aspergillus nidulans*^46^, knocking out the *Ras2* gene either affected the formation of appressoria or caused a variety of morphological defects, such as slower growth and delayed spore germination. In other studies, mutations within Ras either eliminated pathogenicity or caused significant reductions in virulence^36^,^48^,^50^. These observations are consistent with our findings that *Clg2p* was essential for the development and pathogenicity of *C. lunata*.

Based on our findings, we presented a model of the involvement of *Clg2p* in the Ras signalling pathway-mediated appressorium and conidial development and its effect on the pathogenicity of *C. lunata* (Fig. 8). Our results showed that Clg2p physically interacted with *Clf* through its RA domain, which acts upstream of *Clf* to regulate appressorium formation and pathogenicity. Conversely, the interaction of Clg2p-ClUrase altered conidial morphology severely without affecting pathogenicity (Fig. 8). Clg2p is a homologue of the Ras protein family and is known to interact with downstream proteins in the MAPK and cAMP pathways that regulate cellular development and morphogenesis. In *S. cerevisiae*^35^, for example, Ras2p regulates invasive fungal growth by interacting with Cdc42 from the MAPK pathway and Cyr1 from the cAMP pathway. In *F. graminearum,* Ras2 regulates pathogen growth and virulence through the Gpmk1 MAP kinase pathway^36^. The Ras2 of *U. maydis,* however, promotes bud growth by activating the cAMP pathway and regulates morphogenesis by signalling through a MAP kinase cascade^48^. In our study, *Clg2p* was also an important component of the MAPK cascade pathway, and it regulated pathogenicity and the formation of appressoria in *C. lunata*.

We annotated all MAPK pathway homologues in *C. lunata* using information from its genome (unpublished) and identified two key members of the Clf-mediated MAP kinase cascade, MAP kinase kinase and MAP kinase (*Clk1*). This pathway is similar to the Fus/Kss1-MAPK pathway of *S. Cerevisiae* and the *Mst11-Mst7-Pmk1* cascade of *M. grisea*^37^. Interestingly, knocking out *Clf* (*Mst11* homologue) and *Clk1* (*Pmk1* homologue) in *C. lunata* led to a failure to produce conidia, suggesting that they play roles in regulating asexual reproduction and in the production of conidia. By contrast, *Mst11* and *Pmk1* deletion mutants could produce conidia and failed to form appressoria^37^. This novel finding suggests that the MAPK pathways in these two fungal pathogens have slightly different roles. These results will direct future experimentation designed to reveal the regulatory mechanism of conidiation by the *Clf*-mediated MAPK pathway in *C. lunata*.

Our identification of the protein urase oxidase and how its interaction with the Clg2p protein affected conidial morphology has not been reported in previous studies. However, urase oxidase was identified in the interaction between *Fusarium oxysporum* and tomato. Urase oxidase can catalyse the conversion of uric acid to allantoin, forming NH_4_^+^ ions that may be a nitrogen source for the fungus^51^. In microorganisms that infect humans, the ability to hydrolyse urea is linked to several consequences, such as formation of urinary stones in humans pyelonephritis, peptic ulceration and hepatic encephalopathy^52^. Interestingly, *C. lunata* is reported to cause human respiratory tract, skin and corneal infections^53^,^54^. Therefore, we hypothesized that deletion of the *C. lunata* urase oxidase gene affected the utility and absorption of uric acid and altered conidial morphology.

In conclusion, we identified and characterized the *Clg2p* gene in *C. lunata* and found it to be similar to the Ras gene in other phytopathogenic fungi. We proposed its participation in appressorium formation, conidial morphology, and pathogenicity. In addition, we demonstrated participation of the Clf-MAPK cascade in conidiogenesis by the lack of conidia formation in the *Clf* and *Clk1* mutants. Further investigation is required to define the regulatory mechanism of conidiation in this pathogen. The results of this work may provide target sites for designing new chemicals to manage *C. lunata* and similar fungi.

## Materials and Methods

### Fungal strains and culture conditions

*C. lunata* CX-3 provided by Prof. Chen (Shanghai Jiaotong University, Shanghai, China) was used as the wild-type strain and was maintained on potato dextrose agar (PDA) at 28 °C. The selective medium was supplemented with either 250 μg/ml of hygromycin B (50 mg/ml, Roche Applied Science) or 200 μg/ml of Neomycin (Sigma-Aldrich Co. LLC) according to the selection marker gene.

### Cloning full-length cDNA and DNA of *Clg2p*

In our blast analysis, the EST identified as *Clg2p* showed high similarity with *Ras2p* from *Pyrenophora tritici-repentis* Pt-1C-BFP, which was 480 bp in length. To obtain the full-length cDNA sequence of the *Clg2p* gene, 5′-RACE and 3′-RACE were performed using SMART^TM^ RACE cDNA Amplification Kits (Clontech, CA, USA) according to the manufacturer’s instructions. For 5′ RACE PCR amplification, the first and second round PCR reactions were amplified with ClR-5 and UPM, NUP respectively. The cDNA 3′-end was obtained using ClR-3 and a 3′ site adaptor primer. To determine the nucleotide sequence of genomic DNA corresponding to *Clg2p* cDNA, one specific primer pair (1F/R) was designed according to the identified ORF of *Clg2p*. The PCR product was sequenced after cloning into the pMD19-T vector.

### Sequence, phylogeny, copy number and expression analysis of *Clg2p*

Nucleotide and deduced amino acid sequences were analysed using ClustalX1.83 software. Molecular weight determination and isoelectric point predication were conducted by ExPASy. Compute pI/Mw tool (http://www.expasy.ch/tools/pi_tool.html), and conserved domais of the deduced protein were determined using the ScanProsite tool. The phylogenetic tree was constructed using the neighbour-joining method. To assay gene copy number in the genome and confirm the deletion mutants, Southern blotting was performed as previously described by Liu *et al*.^55^.

Real-time quantitative PCR was used to analyse *Clg2p* gene expression at different growth stages as previously described by Liu *et al*.^56^. Primers 2F/R were used for *Clg2p* gene amplification. *GAPDH* was used as an endogenous control. The relative quantitative transcripts were calculated using the 2^−ΔΔC^t method.

### *Clg2p* gene replacement, mutant and complementation

To replace the *Clg2p* gene, first, cassette polymerase chain reaction was used to rescue two flanking sequences of the *Clg2p* gene and was performed using the LA PCR *in vitro* Cloning Kit (Takara Bio Inc., Japan) according to the manufacturer’s instructions. Briefly, a DNA sample from CX-3 was digested with *Eco*RI, *Hin*dIII, *Pstetc*I, *Sal*I and *Xba*I restriction enzymes. The digested DNA was ligated to corresponding sites in the cassette (Takara Bio Inc., Japan). The cassette primers provided in the cloning kit and specific nested primers (3F/3R and 4F/4R) were used to amplify the upstream and downstream sequences of *Clg2p*. The deletion vector was constructed based on the two flanking sequences of *Clg2p*. A 1090-bp upstream fragment of *Clg2p* in the *C. lunata* genome was amplified with primers 5F/R and cloned between the *Hin*dIII and *Xba*I sites on Δ1300 plasmids, resulting in the p1300 g1 vector. Then, a 1094-bp downstream fragment of *Clg2p* was amplified with primers 6F/R and cloned between the *Bam*HI and *Eco*RI sites on the p1300 g1 vector to create a *Clg2p* gene deletion vector (p1300 g11). The ATMT method was used to create the transformants and was performed as previously described by Liu *et al*.^57^.

A 2.112-kb fragment containing the 1.090-kb native promoter and ORF of *Clg2p* was amplified using primers 7F/R and was inserted between the *Hin*dIII and *Bam*HI sites of pCAMBIA1300th, resulting in the complementation vector pCAMBIA1300th-Clg2p. The vector pCAMBIA1300th-Clg2p was co-transformed with the *Clg2p* deletion mutant followed by screening for Neomycin resistance.

### Yeast two-hybrid screening and sequence analysis

Yeast two-hybrid (Y_2_H) screening was performed using the Matchmaker™ GAL4 Two-Hybrid System 3 & Libraries User Manual (Clontech). First, the open reading frame (ORF) of *Clg2p* was amplified with primers 8F/R and inserted into the *Eco*RI and *Bam*HI sites of the pGBKT7 yeast vector (Clontech) to create a bait. An AD fusion library in pGADT7-rescuing cDNA from *C. lunata* was constructed and transferred into the yeast strain AH109 as described in Jing *et al*.^58^. The SD/-Ade/-His/-Leu/-Trp/X-α-Gal medium was used to screen and confirm expression. Then, plasmid DNA from the positive clones was isolated from yeast, transformed into *E. coli* and confirmed by sequencing.

Sequences from positive clones were analysed using BLASTx (http://www.ncbi.nlm.nih.gov). The predicted ORFs of positive colonies were extracted from the genome of *C. lunata* (http://www.ncbi.nlm.nih.gov/nuccore/JFHG00000000) and confirmed by RT-PCR with cDNA as a template. The percentage of similarity and identity of the known sequences were determined using the MatGAT programme. The conserved domains of the deduced proteins were analysed by CDD Search (http://www.ncbi.nlm.nih.gov/Structure/cdd/wrpsb.cgi) and Pfam (http://pfam.xfam.org/).

### Target yeast hybrid assays

To confirm one-to-one interaction, the pGBKT7-Clg2p plasmid was used as bait. The ORF of *Clf* amplified using primers 9F/R, the SAM domain of *Clf* amplified using primers 9F and 10R, the RA domain of *Clf* amplified using primers 11F/R and the ORF of *ClUrase* amplified using primer 12F/R were inserted into the *Nde*I and *Bam*HI sites of the yeast vector pGADT7 (Clontech) to create prey.quadruple dropout medium with -Trp/-Leu/-Ade/-His/X-α-Gal/AbA (QDO/X/A) that was used to detect the interaction. Co-transformants containing (pGBKT7-53+pGADT7-T) and (pGBKT7-Lam+pGADT7) vectors were used as positive and negative controls, respectively.

### Pull-down and Western blotting

The ORF of *Clg2p* was amplified with primers 13F/R and inserted into pGEX-6P-1 to create a GST-Clg2p bait protein. The ORFs of *Clf* and *ClUrase* were amplified using primers 14F/R and 15F/R, respectively, and inserted into pET-28a vectors to create a His-Clf/ClUrase prey protein. The BL21 (DE3) strain was used to express the protein. *In vitro* pull-down assays were performed following the manufacturer’s instructions (Pierce™ GST Protein Interaction Pull-Down Kit (#21516, http://www.thermoscientific.com)). The bait and prey were eluted from 25 μL glutathione agarose. Pull-down proteins were then resolved by 12% SDS-PAGE and detected by Western blotting. GST-Clg2p and HIS-Clf/His-ClUrase fusion proteins were detected by immunoblotting with 1:2,000 diluted anti-GST antibody (#10000-0-AP, PROTEINTECH, http://www.ptgcn.com) and 1:1,500 diluted anti-His (#10001-0-AP, PROTEINTECH) antibody (Millipore), respectively. The goat anti-rabbit IgG (peroxidase conjugate) secondary antibody (Boster, http://www.boster.com.cn) was detected using DBA Western Blotting Substrate (Boster, http://www.boster.com.cn) following the manufacturer’s protocol.

### Bimolecular fluorescence complementation assays

For BiFC assays, ORF of *Clg2p,Clf* and *ClUrase* were amplified with primers 16F/R, 17F/R and 18F/R, subcloned into pSPYNE-35S/ pSPYCE-35S (for split YFP N/C-terminal fragment expression) to form the fusion vector Clg2p-YNE/Clg2p-YCE,Clf-YCE/Clf-YNE and ClUrase-YCE/ClUrase-YNE. The pair of fusion proteins were transiently co-expressed into onion epidermal cells by the GV3101 strain (*Agrobacterium tumefaciens*) transfection as was described for subcellular localization of *Clg2p* genes, with transformed Clg2p-YNE, Clg2p-YCE, Clf-YNE, Clf-YCE, ClUrase-YNE and ClUrase-YCE. A confocal laser scanning microscope (Zeiss LSM510, Germany) was used to detect the fluorescence signal. Co-expressions of target genes and pspYNE or pspYCE were used as negative controls experiments.

### *Clf, ClUrase* and RA domain of *Clf* deletion (*Clf* ^ΔRA^)

The double joint PCR technique was employed to construct genes deletion cassettes for *Clf* and *ClUrase*. The upstream and downstream sequences of both of genes were amplified based on the genome sequence of *C. lunata*. For *Clf* gene deletion, a deletion cassette containing the two flanking sequences of *Clf* surrounding hygromycin B phosphotransferase (*hph*) gene as a selectable marker was amplified by third round PCR described as Yu *et al*.^59^. In first-round PCR, three fragments including upstream fragment of *Clf*, downstream fragment of *Clf* and selectable marker (*hph*) with TrpC promoter of *Aspergillus nidulans* were amplified using primers C1F/C1R, C2F/C2R and Hph-F/Hph-R respectively. In second-round PCR, two fusion cassettes were obtained by fusion PCR technique. The first fusion cassette including the upstream fragment of *Clf* and selectable marker gene was amplified using primer C1F/Hph-R. The second fusion cassette including the downstream fragment of *Clf* and selectable marker was amplified using primer Hph-F/C2R. In third round PCR, the deletion cassettes was amplified using primer C1F and C2R. For *ClUrase* gene deletion, the deletion cassettes were generated with C3F/R and C4F/R described as above. These deletion cassette products were introduced into protoplasts of CX-3 described as Liu *et al*.^55^. Hygromycin resistant transformants were isolated and selected, and used to detect the *hph* gene using primers Hphts and Hphta, and the target gene using primer Clf-F and Clf-R by PCR. To generate the RA domain of *Clf* deletion (*Clf*^ ΔRA^), a 2.5-kb fragment containing the SAM domain and native promoter, 1.5-kb of *Clf* amplified with primers SAM-F/R was cloned between *Bam*HI and *Xba*I sites of pCAMBIA1300th as pCAMBIA1300th-SAM. A 1.3-kb fragment containing protein kinase domain of *Clf* amplified with primers KI-F/R was cloned between *Xba*I and *Hin*dIII sites of pCAMBIA1300th-SAM as pCAMBIA1300th-SAM-K. The vector pCAMBIA1300th-SAM-K was co-transformed into protoplasts of the *Clf* gene deletion mutant described as Liu *et al*.^55^, followed by screening for Neomycin-resistance. All primers in this study are listed in Table S2.

### Analysis of vegetative growth rate, conidiation, conidial and appressorial morphology and infection assays

The analysis of vegetative growth rate and conidiation were as described by Gao *et al*.^9^. Conidial morphology was observed using a light microscope. Three independent experiments were performed with 3 replicates each time, and 100 conidia were observed in each replicate. To observe appressorium structure and position, the conidia were inoculated onto artificial cellophane lying flat on PDA medium and examined under an Olympus Microscope BX51 (Olympus, Center Valley, PA, USA) at 6, 9 and 12 h respectively. Images were photographed using Olympus cellSens^TM^ standard software.

For infection assays, conidial suspensions (1 × 10^6^ conidia/ml in 2% glucose and 0.02% Tween 20 solution) or mycelial plugs were used in sprays or inoculated separately on detached leaves. Three independent experiments were performed with five replicates each time.

## Additional Information

**Accession codes:** All genes in this manuscript had been submitted to GenBank. The gene accession numbers are Clg2p (HQ655805), Clf (KT336108), and ClUrase (KT336109).

**How to cite this article**: Liu, T. *et al*. Clg2p interacts with Clf and ClUrase to regulate appressorium formation, pathogenicity and conidial morphology in *Curvularia lunata. Sci. Rep.* **6**, 24047; doi: 10.1038/srep24047 (2016).

## Supplementary Material

Supplementary Information

## Figures and Tables

**Table 1:** Phenotype of the mutant compared with the wild-type isolate CX-3 (WT).

Strain	Growth rate (mm/day)^I^	Conidiation (10^6^ conidia/ml)^II^	Percentage of crescent- shaped conidia (%)III	Lesion areas (5 cm leaf tips)^IV^
CX-3 (WT)	7.45 ± 0.05a	7.37 ± 0.35a	90.56 ± 3.0a	174.41 ± 5.57a
Δ*Clg2p*	7.05 ± 0.12d	5.41 ± 0.32c	36.1 ± 2.02d	42.49 ± 4.23d
Clg2p-Com	7.22 ± 0.09c	5.81 ± 0.42b	81.56 ± 3.47b	173.89 ± 6.89a
Δ*Clf*	7.01 ± 0.08d	0 ± 0d	–	105.71 ± 7.37c
*Clf*^ΔRA^	7.31 ± 0.08b	5.5 ± 0.18c	81.33 ± 3.80b	113.57 ± 5.59b
Δ*ClUrase*	7.39 ± 0.05ab	5.94 ± 0.43b	43.33 ± 3.00c	171.90 ± 4.72a
Ect	7.32 ± 0.12b	5.86 ± 0.31b	82.67 ± 2.60b	172.21 ± 5.28a

^I^Growth rate was measured on PDA medium.

^II^Conidial numbers harvested from a PDA plate (diameter = 9 cm) after a 12-d incubation at 28 °C.

^III^Conidial morphology is expressed as the percentage of conidia that are crescent-shaped.

^IV^Lesion areas of detached wounded leaves.

All data in all columns are the means of three independent experiments with standard deviations. The statistical analysis was performed using the SAS statistical package (Cary, North Carolina, USA). Statistically significant analysis of variance was further analysed using least significant difference tests. Different letters in each data column indicate significant differences at P = 0.05.
