# Supplementary material for: Clg2p interacts with Clf and ClUrase to regulate appressorium formation, pathogenicity and conidial morphology in Curvularia lunata
Source: Sci Rep. 2016 Apr 4;6:24047. doi: 10.1038/srep24047 (PMC4819193; doi:10.1038/srep24047)
Supplement: Supplementary Information [file srep24047-s1.pdf]

Clg2p interacts with Clf and ClUrase to regulate appressorium formation, pathogenicity and conidial morphology in *Curvularia lunata*

Tong Liu[1, 2]*, Yuying Wang[1], Bingchen Ma[1], Jumei Hou[1], Yazhong Jin[1], Youli Zhang[1], Xiwang Ke[3], Lianmei Tai[1], Yuhu Zuo[1]* & Kishore Dey[4]*

1. Institute of Plant Pathology and Applied Microbiology, School of Agronomy, Heilongjiang Bayi Agricultural University, Daqing, Heilongjiang, 163319, P. R. China

2. State Key Laboratory of Crop Stress Biology in Arid Regions, Northwest A & F University, Yangling, Shanxi, 712100, P. R. China

3. National Coarse Cereals Engineering Research Center, Daqing, Heilongjiang, 163319, P. R. China

4. Department of Plant and Environmental Protection Sciences, University of Hawaii at Manoa, 3190 Maile Way, Honolulu, HI, 96822, USA

*Correspondence authors address: Institute of Plant Pathology and Applied Microbiology, Heilongjiang Bayi Agricultural University, Daqing, Heilongjiang, 163319, P. R. China or Department of Plant and Environmental Protection Sciences, University of Hawaii at Manoa, 3190 Maile Way, Honolulu, HI, 96822, USA

Tel: 86-0459-6819182

Fax: 86-0459-6819182

E-mail: liutongamy@sina.com

Inventory of Supplemental Information

a) Supplemental Tables

b) Supplemental Figures and Legends

Supplementary Tables

Table S1 Positive clone sequence interaction with Clg2p by Blastx analysis

Number	cDNA (bp)	Homologous protein	Blast Score	E-value	homology	function
**1**	1142	Acetyl-coenzyme A synthetase	648	1e-176	Id. 99%; Po. 99%	Catalytic function
**2**	1028	Zinc finger transcription factor ace1	449	5e-149	Id. 70%; Po. 77%	Transcriptional regulation
**3**	577	Uricase	212	2e-64	Id. 85%; Po. 93%	Oxidation reduction
**4**	770	MAPKKK protein STE11	451	1e-148	Id. 95%; Po. 96%	Signal transduction
**5**	1137	Cell wall-associated hydrolase	194	1e-53	Id. 98%; Po. 100%	hydrolysis
**6**	1038	hypothetical protein	443	2E-148	Id. 82%; Po. 92%	unknown
**7**	1038	hypothetical protein	450	2e-156	Id. 90%; Po. 93%	unknown

Table S2 Primers used in this study

Primer name	Sequence 5` to 3`	Purpose
ClR-5	CAATCACCCGTTGGCTCTGC	5`- RACE for *Clg2p*
ClR-3	TCCGAGGAGCCGAAGCAAGG	3`-RACE for *Clg2p*
1F	ATGGACGAGAACAACAGGATAACGA	Amplification ORF of *Clg2p*
1R	CTACCAGCATTTAAGTTTACTCCAG	
2F	GGTTGATTTGGGTTGGAC	Expression of *Clg2p* by Q-PCR
2R	GACGGCAACAACCTGACT	
3F	GTGCGGTGTCAGGGAGAA	Cloning the upstream of *Clg2p* by LA-PCR
3R	ATGTTGGCGACCTCGTATT	
4F	CCGAGGGCAAAGGAATAG	Cloning the downstream of *Clg2p* by LA-PCR
4R	ACAACACCAAGTTATCGCAGT	
5F	CCAAGCTTGTCAAGCCACCCTATCAATC	Amplification of upstream of *Clg2p* and used to construct the p1300g1vector
5R	GCTCTAGAATCCTGTTGTTCTCGTCCAT	
6F	CGGGATCCTGGAGAAACGCTGGTAGGG	Amplification of downstream of *Clg2p* and used to construct the p130011 vector
6R	CGGAATTCCATAGTGAAAGCCACCGAAT	
7F	CCAAGCTTGTCAAGCCACCCTATCAATC	Construct the pCAMBIA1300th-Clg2p vector and used for gene complementation
7R	CGGGATCCAAAGTGGGAGGTTTCGTG	
8F	CGGAATTCATGGACGAGAACAACAGGATAACGA	Construct the pGKT7-Clg2p
8R	CGGGATCCCTACCAGCATTTAAGTTTACTCCAG	
9F	ACACATATGGCCATGCTCGCCTCAAAG	Construct the pGADT7-Clf
9R	TGTGGATCCTTATGCTCCTTGGTTGGTGAT	
10R	TGTGGATCCTTTGTTGCGGAGCTGCTTTGCT	Construct the pGADT7-Clf-SAM
11F	ACACATATGGCTCACGGTCGGAGAAACGCATGT	Construct the pGADT7-Clf-RA
11R	TGTGGATCCTCTTATTCTGACTGCGGTTGC	
12F	ACACATATGTCTCAACTCAGCTACGCT	Construct the pGADT7-ClUrase
12R	TGTGGATCCTTACAGCTTGGACTTGGTTCC	
13F	ACAGGATCCATGGACGAGAACAACAGGATA	form the fusion vector Clg2p-YNE and Clg2p-YCE
13R	TGTGTCGACCCAGCATTTAAGTTTACTCCAG	
14F	ACATCTAGAATGGCCATGCTCGCCTCAAAGT	form the fusion vector Clf-YNE and Clf-YCE
14R	TGTGGATCCGGCTCCTTGGTTGGTGATAAA	
15F	ACATCTAGAATGTCTCAACTCAGCTACGCT	form the fusion vector ClUrase-YNE and ClUrase-YCE
15R	TGTGGATCCCAGCTTGGACTTGGTTCCCTT	
16F	ACAGGATCCATGGACGAGAACAACAGGATA	Construct GST-Clg2p fusion expression protein vector
16R	TGTGAATTCCCAGCATTTAAGTTTACTCCA	
17F	ACAGAATTCATGTCTCAACTCAGCTACGCT	Construct His-ClUrase fusion expression vector
17R	TGTAAGCTTCAGCTTGGACTTGGTTCCCTT	
18F	ACAGAATTCATGGCCATGCTCGCCTCAAAG	Construct His-Clf fusion expression vector
18R	TGTGGATCCTGCTCCTTGGTTGGTGATAA	

C1F	TCGAAGCAGGAGACGAAGTG	
C1R	CATTGATGTGTTGACCTCC<br>TGCGAGTGAGGGTGAAAGG	Amplification of upstream of *Clf* gene
C2F	CGAGGGCAAAGGAATAGAGTAG<br>TACGGTGTTTGGCAGTCC	Amplification of downstream of *Clf* gene
C2R	AACAGGCTTTGACGCATT	
Hph-F	GGAGGTCAACACATCAATG	Amplification of *hph* gene with promoter
Hph-R	CTACTCTATTCCTTTGCCCTCG	
Clf-ZF	CGCCAGCCTCAACTCGTA	Used for hybridized probes for detecting *Clf* gene
Clf-ZR	CCGTCCTCCATCGTAAAT	
C3F	CTGTCGGCAACGGTAATG	Amplification of upstream of *ClUrase* gene
C3R	CATTGATGTGTTGACCTCC<br>GTTAGATGGAGCAGTCACAA	
C4F	CGAGGGCAAAGGAATAGAGTAG<br>GCTCACAAATGCGTCTC	Amplification of downstream of *ClUrase* gene
C4R	AATGTATCGGAACAAGGAA	
GAPDH1	GACGGCAACAACCTGACT	Used as an internal control
GAPDH2	CAGTGCTGCTGGGAATGA	
Hphts	AACTCACCGCGACGTCTGTC	Used for hybridized probes for detecting *hph* gene
Hphta	TTGTCCGTCAGGACATTGTT	
Clg2ppds	TCCGATGCCTTCCTCCT	Detection of *Clg2p* expression to analyze the mutant
Clg2pda	CAGTGCGGTTCACAGACG	
Clf-F	GGTTGGCGACTGGTTGAA	Detection of *Clf* expression to analyze the mutant
Clf-R	TGTCCCGTGAGTGAAGGTAT	
ClU-F	GACCGACACGCAGAAGCAGA	Detection of *ClUrase* expression to analyze the mutant
ClU-R	GTGGCGTCTTTGCCAGTGTT	
ClUts	CAGACAACTAACACGACCCA	Used for hybridized probes for detecting *ClUrase* gene
ClUta	TGCGAATACAAACGGAAG	
SAM-F	CGGGATCCGTCATTGTCTATGCTTTCACGG	Amplification of SAM domain with native promoter and ligated with pCAMBIA1300th
SAM-R	GCTCTAGA GGTGTTGTTCTTTGGTTTCGT	
KI-F	GCTCTAGA CCCTCTTCGTCCCTTGTCAGT	Amplification of kinase domain and ligated with pCAMBIA1300th-SAM
KI-R	CCAAGCTT TTATGCTCCTTGGTTGGTGAT	
RA-F	GCGTGTCATTTACGATGGAGG	Detection of RA domain of Clf
RA-R	TCTTATTCTGACTGCGGTTGC	
SAM-S	CTCGCCGCACTTCAAACAGG	Detection of SAM domain and RA domain of Clf
SAM-A	ATCGGACTCCTCGCCGTCTTCA	
Kinase-F	CCCTCTTCGTCCCTTGTCAGT	Detection of kinase domain of Clf
Kinase-R	TTATGCTCCTTGGTTGGTGAT	
G418F	ATGATTGAACAAGATGGATTGCACG	Detection of neomycin resistance gene
G418R	CGGCGATACCGTAAAGCAC	

Supplementary Figures

Supplementary Figure 1

1 CGCAACTCGCGAGACAAAGGCATAGCATACTGGCAACGAGAGAGCGCAAGAGGCGGACGCC

62 ATGGACGAGAACAACAGGATAACGATTACTGTGTGCGGCGACGGAGGATGCGGTATGGCGCAAACAT

M D E N N R I T I T V C G D G G C G

129 ACATGGAGACTTGAAGGGGGCTCGCAGGCTAATGTGCGCGCAGGAAAGAGTTCAATCACATTGCG

KS S I T L R

194 GCTGGTGAGAAGCGAATGGACATCAGAGTAAGTTGCTCGCGCCCTAATGCGAAGCTGCGTAGGCT

L V R S E WT S E Y

259 TGCAGCGAAAGAGGAATACATTGGCTAACATGTGATGTTCTAGATATGATCCCACAATAGGTACGGC

326 ACGCGCGCGCAACCACTTCATGCTCACGCAGATCTGACTCCCTCTGCAGAAGACTCATACTCAGTAA

D P T I E D

393 CACGTACGATCGACGGCGTCCCTTACTATCTCATGCTCACCGACACCGCTGGCCAAGAAGAATACCG

S Y S V T RT I D GV PY Y L M LT D T A G

460 CGGCCTCTGGGCGGCGTCGAATCTGCAATCCGATGCCTTCCTCCTCGTCTACGACATCACTGCGGCC

Q E E YRG LW A A SN L Q S D A F L L VY

527 AACTCGCTCGACGCACTCGACTACTTCATGGAGATGATTGACATGGAGACGGAGAACCGACTAGAC

D I T A AN S LD ALDY F M E MI D M E T E

593 AATGGAAAAATACCGCCAATCAAGTGTGTGGTCGGCAACAAGTGCGATCTGCAGAGCCAACGGGTG

N RL DN G K I P PI K CVV G N K C D L Q

659 ATTGAAGCAAAGAAAGGCCTCGAGTGGGCACGGAAACGGAAATGCGGCTTCATGGAGACGAGTG

SQ RV I E A K K G L EW A R K R K C G F

723 CGAGAGAAATGGTCAACATTGAAGAGACATTTGCCCGTAAGTCTTTTCTCCCTCCGTCCCATATTCTC

MET S A R E M V N I E

791 TCAGATGGCGGGACCGAGCAATTGCTAACCTGCCCGCGACCAACAGTTCTCGTCCGCCGCGTCGTA

ET F A L LV

857 GAAGCACGCAGACTTACTGCAAACGAGACGTCTGTGAACCGCACTGCTGCCCTTCCCATGTCCAACC

RRVVE A R R LT A N E T S V N R T A A LP

924 CAAATCAACCTGCAGCAAATAACTACAGCGAAAAGCACGATTCCGAGGAGCCGAAGCAAGGCTTCT

MS NP NQPA A N N YS E K H D S E EPKQ G

990 GGAGTAAACTTAAATGCTGGTAGTTTTTCGGTTTAAACCCGGCTCCTCTTAAACTGTCGACATGCTAC

FW S K LK CW

TGCGCAACCCGCAGGCTTCCTACACACACGAAACCTCCCACTTTGGAGGGGCAAATGATGGTGGAA
AATCACGGCCCTTTTTGCTGGAGGAACGCTGGTAGGGAGGGGTACCCAGGCATTCACGTGCGCGCTTCAA
GTTTGTGCGGGCGCGTTGCATCTCAAAATAACTTTCATGACGTTCGCTCCACGGCCAGCTGTCTAGTTTCCCT
TTGTTGTTTTTTCTTCTTGTTTGTTTTATAGCTGCATTTATGCTAGTCACCCTCTGGGTGATTTCCGTCTAGCAG
GAGTATCTGCAAGCGTCCAGTTGACACTCATCTCTCTGCTTTTTCTGTATCTCGTCATGTCTGTTACTAGTGTT
GTTTATTTTCTTTTATCGTTGGATCGGCGTTGAATTGGCGTATGAATTGCTTCCATCTGTTGGTAGCGGAATCG
ACAGGGATCACGGGGTATACCGATACCAGGTGGAAATCTGATTCTTTTGTTCTAAAAAAAAAAAAAAAAAA

Supplementary Figure 1. Gene structure characteristics of *Clg2p*

The *Clg2p* contain 951 nucleotides including 4 exons (marked in blue) and 3 introns. 5`-UTR and 3`-UTR of *Clg2p* are 61 bp and 549 bp with a poly A tail respectively. The open reading frame encoded a peptide of 225 amino acids.

Supplementary Figure 2

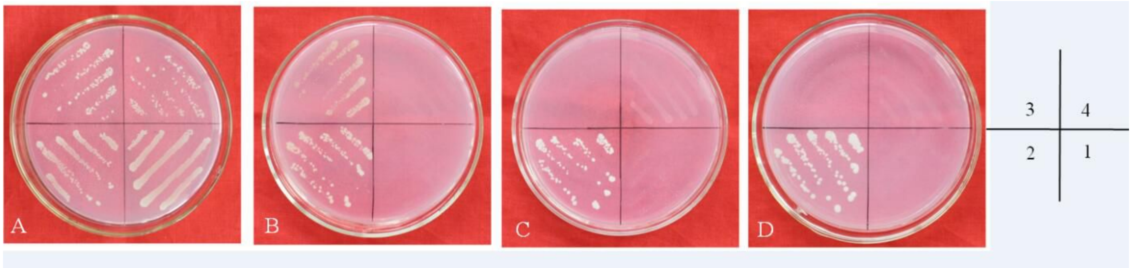

Supplementary Figure 2. Assay of Clg2p protein transcriptional activation on different selection culture media. **A**: SD/-Trp; **B**: SD/-Trp/-Leu; **C**: SD/-Trp/-His; **D**: SD/-Trp/-Ade

Note: 1: pGBKT7-*Clg2p*; 2: positive control; 3: negative control; 4: pGBKT7-*Clg2p*

Supplementary Figure 3

A

MAMLASKSPYPAPSVSGGASQSSSQYNASLNSYRPHASSTRADQSMFASPTESEFSEIYDAPDAIKHWDEDKVGDWLKRINCAQ
YVELFKLNHINGENLMEMDQTHLKDMGIKKVGDRVRIGTQAKQLRNKEYKKASRRTSNRQSLATLDNAAYTPPSSGSPRPLHS
ARSNPPLSNPVSSRSEKRMSRQITNSDLSSYAYGTKSPSRPGSPLVDQETRNLRSQRQGGMNSPKDGGNKTYGAHPLSASSSSANI
ASAHARNQRTTPTETPQTARFAHHVRASPSMDSATNSAILPHDKALVRVIYDGGRTSVVNIEGCKTAEEVMLKTLTKGHLNVAHV
KNYCFYILNSPEPDPSLSERLSDIEVFRLVKDANRHERGRLILRKVHAGEPDEDQLKAAAGIYQQQNYQQPQNVYVPTSNRSQN
KIEKLTGESLAAVSYPLSPTSARERERHINSTAQNLEGPAEASRSPYFQARARKLKQFYGARPPSELITSDLTSYFPDVGKDEIDKT
VRMSIRRSHRLSRAASRLSMASNFSVASSLKDAPPLPSIADSWLQGGAQARPLRPLSVMRLGLPHQSGYRDSLASSVLEPLDEES
PLEPNRKSYVSFGGDSVGDSLAITDPDGNTTLQSYFDDAGSSLAGSSSSNTENGDSLNKRLSEALAEDGEESDDELAEYLEQDSW
DNVKYMKGALIGQGSFGSVYLALHAVTGELMAVKQVELPSVAGASQMDHKKTNMVEALKHEIGLLRELKHKNIVQYLGSNSD
ESHLNIFLEYVPGGSVATMLVNYGPLGESLIQNFVRQILTGLSYLHSRDIIHRDIKGANILVDNKGSVKISDFGISKRIEASTLGGSK
KGAQRVSLQGSVFWMAPEVVRQTAYTRKADIWSLGCLVVEMFTGSHPHPNCTQLQAIFKIGGSGDASPTIPDNAGEDARKFLAE
TFLIDHEKRPSADDLLASSFITNQGA

B

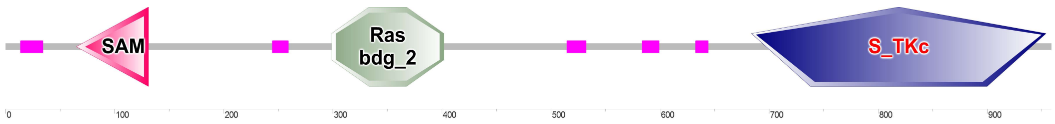

Supplementary Figure 3. Amino acid sequence (A) and protein structure characterization using SMART software (http://smart.embl-heidelberg.de) of Clf (B)

The Clf contain an open reading frame 2880bp, and encoded 959 amino acids, including an sterile alpha motif domain at the N terminus (SAM), a well conserved Ras-association domain (Ras bdg_2) and a serine/threonine protein kinases, catalytic domain (S_TKc).

Supplementary Figure 4

A

1 ATGTCTCAACTCAGCTACGCTCGCTACGGCAAGGACAATGTTCGCCTGTACAAGGTGGACAAGGACCCC

M S Q L S Y A R Y G K D N V R L Y K V D K D P

70 AAGACTGGCATTCACACAGTCGTCGAGCAGACAGTGCGCATCTTGCTCGAGGGTGACATTGAGCAAT

K T G I H T V V E Q T V R I L L E G D I E Q

137 CGTAAGCATGGTATACTAACGCAGATAGCCATCAGACAACTAACACGACCCAGCTACACCAAAGCCGAC

S Y T K A D

206 AACTCAGTGGTAGTCGCGACCGACACGCAGAAGCAGACCACCTACATTCTCGCAAAGCAACACCCTAT

N S V V V A T D T Q K Q T T Y I L A K Q H P I

274 CGATCCACCCGAGCAGTTCGCTGCCATCATTGGCGACCACTTTATCAAGACCTACCCCCATATCCACGC

D P P E Q F A A I I G D H F I K T Y P H I H A

343 CGCCCATGTCAAGATCATCCAGCACCGATGGACGCGCATGACCATTGACGGCAAAGCCCACCCACACT

A H V K I I Q H R W T R M T I D G K A H P H

411 CGTTTTACCGCGACGGCGAGGAGACGCGCGTTATCGAGGCCGTAACCCGAGAGAACGTTGGCGTCTC

S F Y R D G E E T R V I E A V T R E N V G V S

478 CATTCGGTCCAAGATTGAGAAGCTCCTGGTCCTGAAGAGCACGGGTTCAGCATTCCATGGCTTCCATCG

I R S K I E K L L V L K S T G S A F H G F H R

547 CGACGAGTACACCAGACTGCCTGAGACATGGGACCGTATCCTGAGCACCGATATCGAGGCCGGATGG

D E Y T R L P E T W D R I L S T D I E A G W

614 CAGTGGAAGCTATTCAAGGCCTTGGACGAGGTCAAGGCTATCGACTTCAACGCTGCATGGAAAACAG

Q W K L F K A L D E V K A I D F N A A W K T

681 CGAGGGATATCACCATGAAGATTTTTGCCGAGGACGACAGTGCTAGCGTGCAGGCGACCATGTACAA

A R D I T M K I F A E D D S A S V Q A T M Y K

748 GATGTGCGACTTGATCTTGGCTGCGATTCCTGAGGTCGAGGCTGTGGACTACTCGCTCCCTAACAAGC

M C D L I L A A I P E V E A V D Y S L P N K

816 ACTACTTTGAAATTGGTCAGTAACTTCCGTTTGTATTCGCACATTCCAGTTTGCTGACACTATGCCCAGA

H Y F E I D

886 CCTCTCATGGCACAAGGACATTAAGAACACTGGCAAAGACGCCACCGTTCTCGCTCCTCAAACTGACC

D L S W H K D I K N T G K D A T V L A P Q T

954 CCAACGGTCTCATCCAGTGCACCGTTACCCGCAAGGGAACCAAGTCCAAGCTGTAA

D P N G L I Q C T V T R K G T K S K L

B

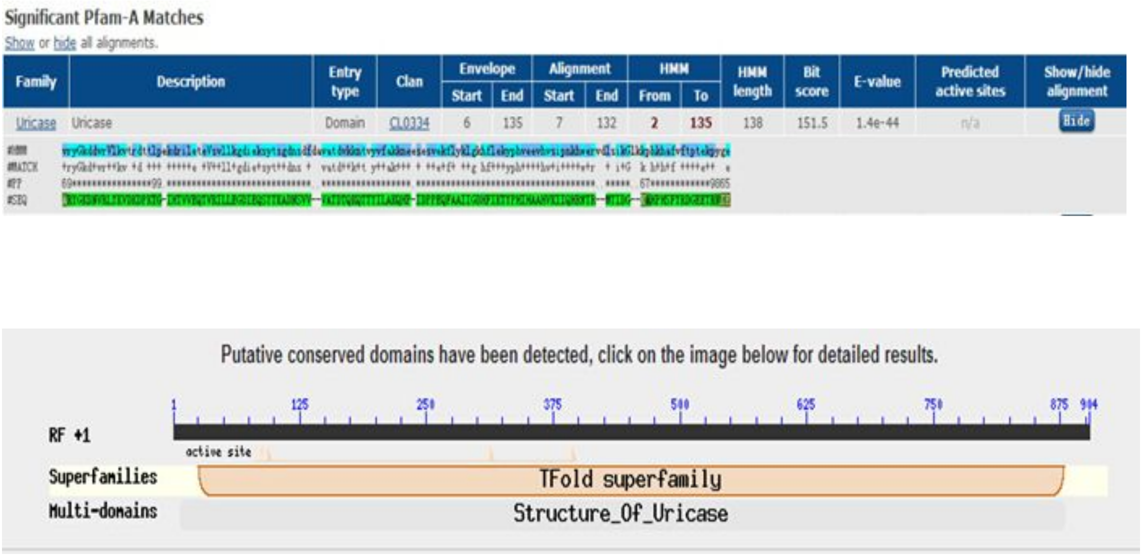

Supplementary Figure 4. Gene and protein structure characteristics of *ClUrase*

The *ClUrase* contain 1009 nucleotides including 3 exons (marked in yellow) and 2 introns. The open reading frame encoded a peptide of 300 amino acids (**A**). The Protein structure characterization of ClUrase using Pfam27.0 (http://pfam.xfam.org/search) and NCBI CDD (http://www.ncbi.nlm.nih.gov/Structure/cdd/wrpsb.cgi) analysis (**B**).

Supplementary Figure 5

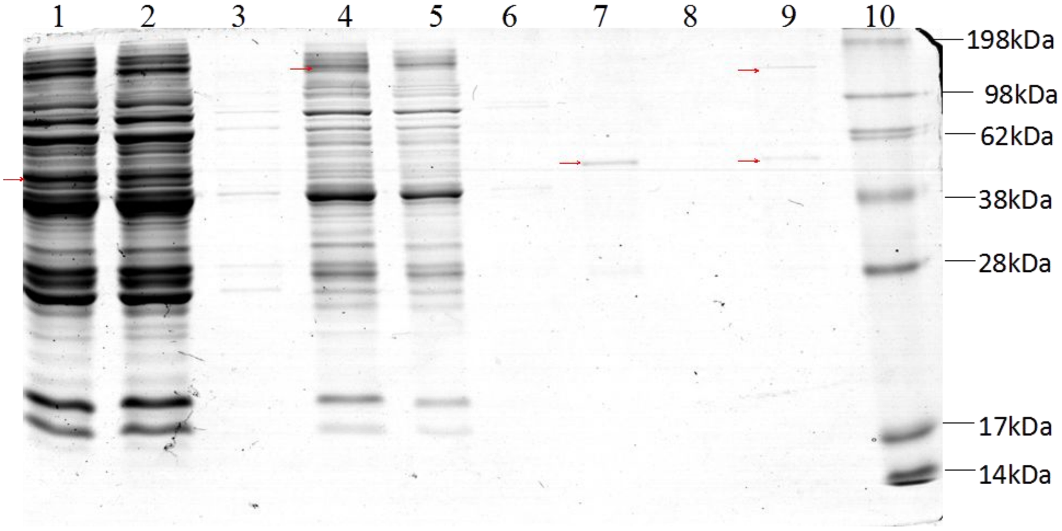

Supplementary Figure 5. A pull down analysis of interaction between Clg2p and Clf

Lane 1: GST-Clg2p (bait) protein lysate; Lane 2-3: GST-Clg2p(bait) protein flow-through and wash; Lane 4: His-Clf (prey) protein lysate; Lane 5-6: His-Clf (prey) protein flow-through and wash; Lane7: purified GST-Clg2p (bait) protein; Lane 8: His-Clf (prey) protein control; Lane 9: purified Clg2p-Clf (bait-pey); Lane 10: protein marker.

Supplementary Figure 6

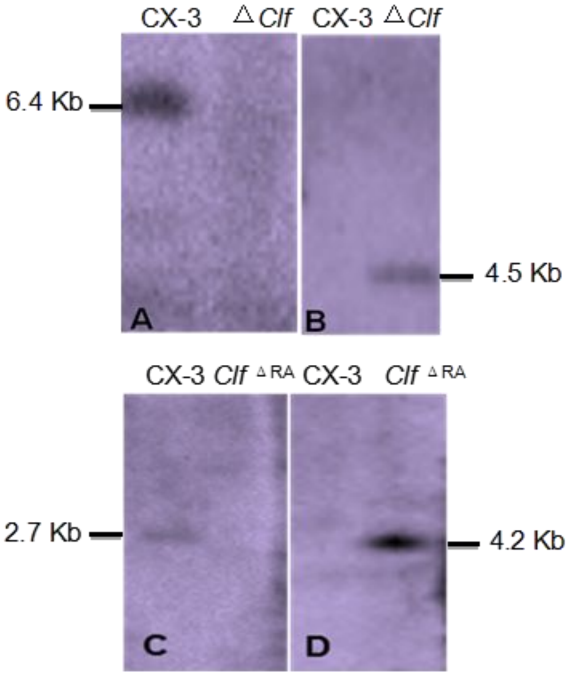

Supplementary Figure 6. Southern blotting analysis of △*Clf* and *Clf*$^{\Delta RA}$

Total of genomic DNA samples isolated from wild-type strain CX-3 and △*Clf* were digested with Hind Ⅲ. A 951-bp PCR fragment amplified from wild-type strain CX-3 with primer Clf-ZF and Clf-ZR was labeled as probe (**A**). A 607-bp PCR fragment of *hph* gene amplified with primer Hphts/Hphta was used as hybridzation probe (**B**). Total of genomic DNA samples isolated from wild-type strain CX-3 and *Clf*$^{\Delta RA}$ were digested with Kpn I . A 371-bp PCR fragment amplified from wild-type strain CX-3 with primer RA-F and RA-R was labeled as probe (**C**). Total RNA samples from wild type strain CX-3 and *Clf*$^{\Delta RA}$ were subjected to were digested with XhoI . A 742-bp PCR fragment of neomycin resistance gene with primer G418F/R was labeled as probe (**D**) .

Supplementary Figure 7

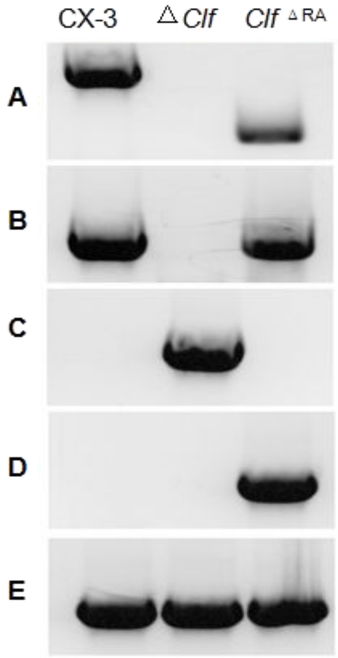

Supplementary Figure 7. RT-PCR analysis of △*Clf* and *Clf*$^{\triangle RA}$

Detection of *Clf* gene using primer Clf-F/R in wild type isolate CX-3, △*Clf* and *Clf*$^{\triangle RA}$(**A**). Detection of SAM and RA domain of *Clf* gene using primer SAM-S/A in wild type isolate CX-3, △*Clf* and *Clf*$^{\triangle RA}$(**B**). Detection of RA domain of *Clf* gene using primer RA-F/R in wild type isolate CX-3, △*Clf* and *Clf*$^{\triangle RA}$(**C**). Detection of neomycin resistance gene using primer G418F/R in wild type isolate CX-3, △*Clf* and *Clf*$^{\triangle RA}$(**D**). Detection of *GAPDH* gene using primer GAPDH1/2 in wild type isolate CX-3, △*Clf* and *Clf*$^{\triangle RA}$(**E**).

Supplementary Figure 8

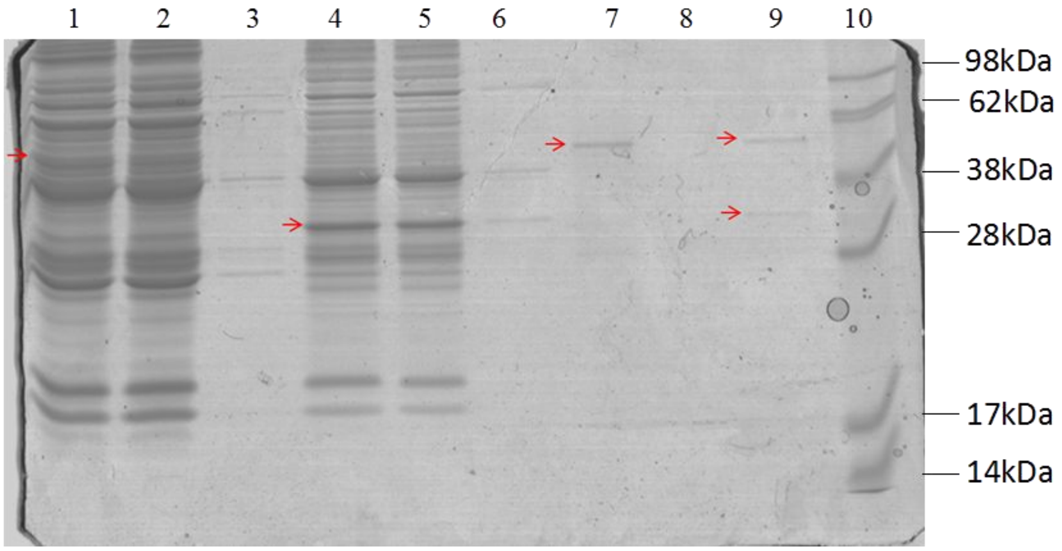

Supplementary Figure 8. A pull down analysis of interaction between Clg2p and ClUrase

Lane 1: GST-Clg2p (bait) protein lysate; Lane 2-3: GST-Clg2p (bait) protein flow-through and wash; Lane 4: His-ClUrase (prey) protein lysate; Lane 5-6: His-ClUrase (prey) protein flow-through and wash; Lane 7: purified GST-Clg2p (bait) protein; Lane 8: His-ClUrase (prey) protein control; Lane 9: purified Clg2p-ClUrase (bait-pey); Lane 10: protein marker
